# 

**Published:** 1859-01

**Authors:** 


### LIST OF COLLABORATORS.

S. G. Armor, M.D., late Professor of Pathology and Clinical Medicine, Missouri Med. College.
John L. Atlee, M.D., late President Pennsylvania State Medical Society.
Walter F. Atlee, M.D., Philadelphia.
Robert G. Barclay, M.D., Jerusalem, Syria.
John Bell, M.D., Philadelphia, formerly Professor of Medicine, Medical College of Ohio.
T. S. Bell, M.D., Professor of Medicine, University of Louisville.
S. M. Bemiss, M.D., Professor of Clinical Medicine, University of Louisville.
L. P. Blackburn, M.D., New Orleans, La.
George C. Blackie, M.D., Professor of Botany, University of Nashville.
G. C. Blackman, M.D., Professor of Surgery, Medical College of Ohio.
J. L. Bobbs, M.D., formerly Professor of Anatomy, Indianapolis.
W. M. Boling, M.D., Montgomery, Ala., formerly Professor of Midwifery, Transylvania University, Lexington, Ky.
W. S. Bowen, M.D., New York.
N. Bozeman, M.D., Montgomery, Ala.
J. T. Bradford, M.D., Augusta, Ky.
John H. Brinton, M.D., Lecturer on Operative Surgery, Philadelphia.
W. S. Chipley, M.D., Professor of Medicine, Transylvania University.
A. Clapp, M.D., New Albany, Ia.
D. B. Cliffe, M.D., Franklin, Tenn.
J. Da Costa, M.D., Lecturer on the Practice of Medicine at the Philad. Med. Association.
Samuel H. Dickson, M.D., Professor of Medicine, Jefferson Medical College.
M. Dupierris, M.D., Havana, Cuba.
Richard J. Dunglison, M.D., Philadelphia.
W. F. Edgar, M.D., United States Army.
S. C. Farrar, M.D., Jackson, Miss.
C. S. Fenner, M.D., Memphis, Tenn.
Austin Flint, M.D., Professor of Clinical Medicine, Auscultation and Percussion, New Orleans School of Medicine.
Charles Frick, M.D., Baltimore.
W. Y. Gadbury, M.D., Benton, Miss.
O. C. Gibbs, M.D., Chautauque County, New York.
Dr. J. P. Hall, Glasgow, Ky.
William A. Hammond, M.D., United States Army.

JOHN HARDIN, M.D., Professor of Obstetrics, Kentucky School of Medicine, Louisville.

EDWARD HARTSHORNE, M.D., One of the Surgeons to Wills Hospital for Diseases of the Eye, Philadelphia.

E. B. HASKINS, M.D., Professor of Chemistry, Shelby Medical College, Nashville, Tenn.

C. A. HENTZ, M.D., Marianna, Florida.

ADDINELL HEWSON, M.D., One of the Surgeons to Wills Hospital for Diseases of the Eye, Philadelphia.

SAMUEL L. HOLLINGSWORTH, M.D., late Editor of the Medical Examiner, Philadelphia.

WILSON JEWELL, M.D., Philadelphia, President of the Quarantine and Sanitary Convention.

WM. V. KEATING, M.D., Lecturer on Obstetrics and the Diseases of Women, at the Philadelphia Medical Association.

JOHN G. KERR, M.D., Canton, China.

G. A. KUNKLER, M.D., Madison, Ia.

BEN. LOGAN, M.D., Shreveport, La.

A. LOPEZ, M.D., Mobile, Ala., formerly President of the Alabama State Medical Society.

J. AITKEN MEIGS, M.D., Professor of the Institutes of Medicine in the Philadelphia College of Medicine.

S. WEIR MITCHELL, M.D., Lecturer on Physiology at the Philad. Med. Association.

GEORGE R. MOREHOUSE, M.D., Philadelphia.

B. I. RAPHAEL, M.D., New York.

J. C. REEVE, M.D., Dayton, Ohio.

L. C. RIVES, M.D., formerly Professor of Midwifery, Medical College of Ohio.

J. LAWRENCE SMITH, M.D., Professor of Chemistry, University of Louisville.

W. C. SNEED, M.D., Frankfort, Ky., late President Kentucky State Medical Society.

C. H. SPILMAN, M.D., Harrodsburg, Ky., late President of Kentucky State Medical Society.

GEO. SUTTON, M.D., Aurora, Ia.

W. L. SUTTON, M.D., Georgetown, Ky., formerly President Kentucky State Medical Society.

HORATIO R. STORER, M.D., formerly one of the Physicians to the Boston Lying-in Hospital, Boston, Mass.

S. THOMPSON, M.D., Albion, Ill., late President of the Illinois State Medical Society.

E. WILLIAMS, M.D., Cincinnati, Ohio.

### Bi-Monthly Bibliographical Record.

AMERICAN.

The Physician's Hand-Book of Practice for 1859. By Wm. Elmer, M.D. New York: W. A. Townsend & Co. (From the publishers.)

Some Local and General Excrescences of Homoeopathy. By John T. Geary, M.D. Pamphlet, pp. 47. Philadelphia, 1858. (From the author.)

Fourth Annual Report to the Legislature of South Carolina, relating to the Registration of Births, Deaths, and Marriages, for the year ending December 31st, 1857. Pamphlet, pp. 98. Columbia, S. C., 1858.

Brief Expositions of Rational Medicine: to which is prefixed the Paradise of Doctors. A Fable. By Jacob Bigelow, M.D., etc. 12mo., pp. 69. Boston: Phillips, Sampson & Co., 1858. (From the author.)

The Treatment of the Paralysis of Motion. By Charles F. Taylor, M.D. pp. 15. Reprint. New York, 1858. (From the author.)

An Essay on Inflammation. By J. H. Watters, M.D., etc. Reprint, pp. 32. St. Louis, 1858. (From the author.)

Bradycrote Treatment of Yellow Fever, by Veratrum Viride and Gelseminum Sempervirens. By Drs. White and Ford. Reprint, pp. 7. Charleston, 1858. (From the authors.)

Typhus Fever in Great Britain. By J. B. Upham, M.D., etc. Reprint, pp. 46. Boston, 1858. (From the author.)

Hints to Craniographers, etc. By J. Aitken Meigs, M.D., etc. Reprint, pp. 8. Philadelphia, 1858. (From the author.)

An Essay on the Climate and Fevers of the South-western, Southern, Atlantic, and Gulf States. By James C. Harris, M.D. Reprint, pp. 32. New Orleans, 1858. (From the author.)

Diphtheritis: a Concise Historical and Critical Essay of the Late Epidemic Pseudo-membranous Sore-Throat of California. By V. J. Tourgeaud, M.D. Pamphlet, 8vo., pp. 44. Sacramento, 1858. (From the author.)

Transactions of the Third Session of the Medical Society of the State of California, Convened at San Francisco, February, 1858. 8vo., pp. 168. Sacramento, 1858. (From the secretary.)

FOREIGN.

A Treatise on Venereal Disease. By John Hunter, F.R.S., with Copious Additions, by Dr. Philip Ricord. Translated and Edited with Notes, by Freeman J. Bumstead, M.D., etc. Second edition, revised, containing a résumé of Ricord's recent Lectures on Chancres. 8vo., pp. 548. Philadelphia: Blanchard & Lea, 1858. (From the publishers.)

A Treatise on Fractures. By J. F. Malgaigne. Translated from the French, by John H. Packard, M.D. 8vo., pp. 684. Philadelphia: J. B. Lippincott & Co., 1859. (From the publishers.)

On Vesico-Vaginal Fistula; and its Successful Treatment, Illustrated by Eleven Cases. By I. Baker Brown, F.R.C.S., etc. Pamphlet, pp. 27. London, 1858. (From the author.)

Étude sur la Monorchidie et la Cryptorchidie Chez l'Homme. Par M. Ernest Godard, M.D., etc. 8vo., pp. 164. Paris: Victor Masson, 1858.

On Chloroform and other Anæsthetics; their Action and Administration. By John Snow, M.D., etc. Edited, with a Memoir of the Author, by B. W. Richardson, M.D. 8vo., pp. 444. London: Churchill, 1858.

The Cause of Death in the Still-born. By Dr. King. Second edition. Small 8vo., pp. 68. London: Churchill, 1858.

Traité de l'Angine Glanduleuse, et Observations sur l'Action des Eaux-Bonnes dans cette Affection; précédées de Considération sur les Diathèses. Par Noël Gueman De Mussy. 8vo., pp. 269. Paris: Victor Masson, 1858.

On Amputation by a Long and Short Rectangular Flap. By T. P. Teale, F.L.S., etc. 8vo., pp. 72. London, 1858.

Ophthalmistrik. Nach den neuesten Forschungen für das Studium und die Praxis bearbitet von Carl Herman Schanenberg. Zweite Auflage. 8vo., pp. 278. Lahr: Baden, 1858.

The First Step in Chemistry. By Robert Galloway, F.C.L., etc. 8vo. London: Churchill, 1858.

Nutrition in Health and Disease. By James Henry Bennett, M.D. pp. 210. London, 1858.

On Dropsy Connected with Disease of the Kidneys, (Morbus Brightii,) and on some other Diseases of those Organs Associated with Albuminous and Purulent Urine. By W. B. Basham, M.D., etc. 8vo., pp. 241. London: Churchill, 1858.

Observations on Venereal Diseases, derived from Civil and Military Practice. By Hamilton Sabatt, A.B., etc. pp. 283. Dublin: Fannin & Co., 1858.

Traité d'Anatomie Chirurgicale et de Chirurgie Expérimentale. Par J. F. Malgaigne, M.D., etc. Deuxième Édition. 8vo., pp. 1664. Paris: Balliere et fils, 1858.

Contributions a la Statistique de la Lithotritie. Par le Dr. Auguste Swalin. 8vo., pp. 124. Stockholm, 1858.

